# SARS-CoV-2 transmission risk screening for safer social events: a non-randomised controlled study

**DOI:** 10.1038/s41598-022-16905-w

**Published:** 2022-07-27

**Authors:** Rafel Ramos, Lia Alves-Cabratosa, Jordi Blanch, Àlex Pèlach, Laura Albert, Quirze Salomó, Sílvia Cabarrocas, Marc Comas-Cufí, Ruth Martí-Lluch, Anna Ponjoan, Maria Garcia-Gil, Salomé de Cambra, Albert d’Anta, Elisabet Balló, Albert Alum, Rosa Núria Aleixandre

**Affiliations:** 1 Vascular Health Research Group (ISV-Girona), Institut Universitari d’Investigació en Atenció Primària Jordi Gol (IDIAP Jordi Gol), c/ Maluquer Salvador, 11 baixos, 17002 Girona, Catalonia Spain; 2 Department of Medical Sciences, School of Medicine, Campus Salut, Universitat de Girona, Girona, Catalonia Spain; 3 Biomedical Research Institute of Girona (IdIBGi), Salt, Girona, Catalonia Spain; 4 Centre Blockchain de Catalunya, Parc Científic i Tecnològic de la Universitat de Girona, Girona, Catalonia Spain; 5 Col·legi de Farmacèutics de Girona, Girona, Catalonia Spain; 6 Computer Science, Applied Mathematics and Statistics Department, Universitat de Girona, Girona, Spain; 7 Unitat de Qualitat i Seguretat del Pacient, Atenció Primària, Institut Català de la Salut, Girona, Catalonia Spain; 8 Consell de Col·legis Farmacèutics de Catalunya, Barcelona, Catalonia Spain; 9 Consell Social Universitat de Girona, Girona, Catalonia Spain

**Keywords:** Viral infection, Epidemiology

## Abstract

There is an ongoing debate on the implementation of the COVID-19 passport throughout Europe. We sought to build and test a feasible prevention strategy to ensure low SARS-CoV transmission risk in public events. We conducted a non-randomised controlled study. The intervention group obtained a confidential digital certificate of very low capacity for transmitting SARS-CoV-2 and attended socio-cultural events in Girona (Spain) between 1 April and 21 May 2021. The primary care services and a network of pharmacies cooperated in providing the certification. A group of non-attendees was randomly selected from pseudonymised health records as controls. We estimated the incidences of SARS-CoV-2 infection and recorded the challenges in the process. Follow-up was complete for 1351 participants, who were matched with 4050 controls. Mean age of the study population was 31.1 years, and 53% of participants were women. Incidence rates of SARS-CoV infection at 14 days in the group of attendees and non-attendees were 15.9 and 17.7 per 100,000 person-days, respectively; the difference between incidences was − 1.8 (95% CI − 22.8, 19.3). Implementation problems were minor, and 89.2% of respondents to a survey were satisfied with the process. The incidence rate of SARS-CoV-2 infection was not different in the intervention and control groups. These results are in favour of establishing a COVID-19 certificate to attend public events, and connote feasibility of implementation at a population level.

## Introduction

Severe acute respiratory syndrome coronavirus 2 (SARS-CoV-2) is considered the most severe pandemic of the twenty-first century thus far. Over 190 million confirmed cases and more than four million of coronavirus disease 2019 (COVID-19)-related deaths accumulated in all continents as of 26 July 2021, according to the World Health Organization^1^. Such dissemination was mainly driven by the high transmissibility of this virus.

Measures to restrict mobility and social life are effective at controlling this pandemic, but have had grave consequences on the economic and social fabric^2^, and thus on the population’s wellbeing. Culture, sports, leisure, and catering have been the most affected sectors^2^. Moreover, confinement has shown an association with an increased incidence of pathologies related to mental health^3,4^. At the time this study was carried out, Spain was being struck by a fifth wave with a dramatic increase in the incidence of new infections that eventually reached the vulnerable population, despite vaccination coverage of 30–40% that hindered further escalation of hospitalisation rates. Later on, in July 2020, certain countries in Central Europe reinstated lockdown measures or were planning to do so. Some other areas, like Catalonia (Spain) were discussing the implementation of the EU digital COVID certificate, to allow certain social and economic activities without weakening health security. In this regard, mass-gathering events deserved special attention, because of their association with a high risk of SARS-CoV-2 dissemination^5,6^; this risk was particularly high in sports events and concerts^7^.

Previous studies indicated that mass-gathering events could be safe in the context of a COVID-19 outbreak if a comprehensive preventive intervention was implemented^8–10^. Such evidence includes a randomised trial that examined a large number of participants and had sufficient statistical power to estimate SARS-CoV-2 transmission risk^9^. However, these studies examined one (musical) event. Further confirmation and inclusion of various social events, different scenarios of the pandemic, and vaccinated as well as non-vaccinated people would be of interest. Even more, feasibility of implementation of these preventive measures is crucial and thus should be explored. Accordingly, we sought to analyse whether people who attended public events with a certified low risk of transmitting SARS-CoV-2 infection, through a negative result from a rapid antigenic test (up to 36 h previous to the event) or proven immunisation against SARS-CoV-2 (confirmed vaccination, or confirmed recovery from COVID-19) presented a higher rate of detected SARS-CoV-2 infections compared to a group of people who did not attend such events. We also aimed to frame such analysis into an integrated strategy that could be deployed throughout the territory.

## Methods

### Study design and participants

*ObrirGirona* was a non-randomised controlled study that compared attendees to social and cultural events (intervention group) with a matched group of non-attendees (as controls). The study involved social and cultural events held in the city of Girona (Catalonia, Spain) between 1 April and 21 May 2021. Participants could choose to have dinner at any of the five participating restaurants (on three weekends) or either of the following two concerts: *La Mercè Electrònica*, and a concert tribute to health professionals by *Manel* in *La Mirona*, Salt (Catalonia, Spain). Although the bar services in the concerts were outdoors, both types of events -concerts and dinner at restaurants- were indoors, we evaluated mainly indoor activities.

Invitation to participate in the group of attendees was voluntary and advertised on mass and social media. Individuals from the general population willing to enter the study had to download the free AOKpass application program (app), to create a digital certificate of very low risk of transmission (VLRT), which had to be validated at a pharmacy.

One of the following requirements was necessary to obtain the digital certificate: proof of recovery from SARS-CoV-2 infection, proof of previous vaccination, or negative antigen test. (1) A positive PCR test result for SARS CoV-2 (from three months before up to 15 days prior to the index date) confirmed previous SARS-CoV-2 infection. This result had to be certified by an authorised clinical analysis laboratory and granted the validity of the digital VLRT pass for three months, starting from the date of analysis; (2) proof of vaccination status against SARS-CoV-2 required a standardised form from the Catalan Institute of Health (*ICS-Institut Català de la Salut*). This document certified six months of immunisation. If neither of these two documents could be provided (3) a negative antigen test for SARS-CoV-19 had to be performed at the pharmacy, which guaranteed validity of the digital VLRT pass for 36 h.

Exclusion criteria applied to people with COVID-19 symptoms or with a positive PCR test result within 15 days before the event. An anamnesis inquired about possible COVID-19 symptoms, and addressed the need for quarantine, which was mandatory when there had been previous contact with a person with SARS-CoV-2 infection. Participants also had to fill in and sign a responsibility form where they stated lack of COVID-19 symptoms, or recent contact with a person with SARS-CoV-2 infection. If a person had symptoms (fever, persistent cough, shortness of breath, recent anosmia/ageusia, or two of the following: sore throat, runny nose, fatigue, muscle aches, headache, stomachache with vomiting or diarrhoea) no further testing was performed and that person was directed to the healthcare services and followed the corresponding SARS-CoV-2 protocols in force at the time.

Participants received information on the cost of obtaining the VLRT pass, which included the performance of the test when needed and the creation of a certificate (the cost of a test plus the certificate was 8.5€; the cost of the certificate [of vaccination or laboratory analysis] was 2.5€).

The control group of non-attendees was a random selection from the clinical health records of people assigned to the public primary care services who had not attended the event. Data from this sample was pseudonymised and matched with participants in the intervention group by age, sex, postal code, history of previous SARS-CoV-2 infection, vaccination status, and event date.

We calculated the sample size to estimate the difference between two independent proportions using the ARCSIN approximation. Considering an alpha risk of 0.05 and a beta risk < 0.2 in a bilateral contrast, we should require 1260 people in the first group (of attendees) and 3780 in the second (of non-attendees), to detect a statistically significant difference between two proportions, which were expected to be 0.012 in the first group and 0.0030 in the second group. We estimated a 0% of follow-up loss rate. The matching ratio was 1:3. For this calculation, we considered the cumulated incidence observed before the beginning of the study: 300 per 100,000 person-years^11^ (Supplementary Fig. S1).

The index date was that of the event in both the group of attendees and in the matched group of non-attendees. After data pseudonymisation, follow-up was carried out, through electronic health records from the ICS, and lasted 14 days from index date. No project-specific testing was performed during follow-up.

### Variables

We recorded the following variables from the intervention group of attendees: date of birth, sex, symptoms compatible with COVID-19—exclusion criterion—(yes/no), derivation to a COVID-19 primary care manager (yes/no), test type (if performed), test result. We also recorded whether participants presented proof of SARS-CoV-2 vaccination, or a certified report with a PCR test result.

The pseudonymised data from participants in the control group of non-attendees comprised the following variables: age, sex, postal code, history of previous SARS-CoV-2 infection (yes/no), and SARS-CoV-2 vaccination status (vaccinated/unvaccinated).

Data downloading was carried out by the Quality and Safety Unit from the Clinical Management Area of the Primary Care Directorate in ICS Girona. This Unit performed the data pseudonymisation before delivery to the research team, who conducted the analysis.

### Outcomes

The main outcome was incident SARS-CoV-2 infection, defined by a positive confirmatory test (rapid antigen test, or PCR) in the first 14 days after the social event. Additionally, we collected information on possible logistical and implementation problems during the process of generating the secure pass for the intervention group (attendees). We also collected the opinion and satisfaction level of a subsample of 149 individuals within the first 468 participants from the intervention group. Three questions about global satisfaction, economic cost, and intention of recommending the process were asked to these people. The level of agreement was classified according to a Likert scale. Respondents could select an answer from the following: totally agree, agree, agree a little, disagree a little, disagree, and totally disagree.

### Procedures and circuits

The app AOKpass (developed in partnership with the International Chamber of Commerce—ICC, International SOS, and *Société Générale de Surveillance*—SGS) is a secure, simple, and unequivocal pass to certify that a person has a VLRT in an access control point (managed by non-healthcare professional). This app ensures data privacy and total control over the use of one’s personal information. For each validated user, the app only sends a hash with the data emitted from a mobile phone; user identification is not possible. Personal data of users who acted as certificate generators were not saved, only their username and password.

Users who wished to attend one of the proposed events within the project went to one of the pharmacy offices across Girona, Salt, Sarrià, and Sant Julià de Ramis (Catalonia, Spain) that volunteered to participate in this study. These pharmacies allocated an individual ventilated extraction space and procured all the mandatory protective and waste collection equipment. The entire procedure was coordinated and monitored by the Official College of Pharmacists of Girona (COFGi). The antigen test used in this study was SARS-CoV-2 Rapid Antigen Test^12^, which has high sensitivity and specificity^13^, and analyses nasal swabs -instead of the nasopharyngeal ones- facilitating implementation. Training on all the procedures was carried out by pharmacists and clinical analysts from the COFGi and technical staff from the Blockchain Centre of Catalonia. This program was coordinated by the primary healthcare and the COVID-19 managers in Girona, for referral of positive or suspected cases and later monitoring during the 14 follow-up days.

In the context of the study, access control to the events included reading the AOK pass to certify VLRT, participants had to wear FFP2 masks that could only be removed at the time of drinking or eating in the allocated spaces, no minimum distance was required when attending the events, and the staff working at the events were required to have a negative antigen test for SARS-CoV-2 from the previous 36 hours.

### Statistical analysis

Normal continuous quantitative variables were described with the mean and standard deviation, and qualitative variables with percentages. The comparison of participants’ characteristics between groups was performed using student’s t-test and χ^2^ test for continuous and categorical variables, respectively. We estimated the incidences of SARS-CoV-2 infection at seven and 14 days; and the corresponding 95% confidence intervals (CI) using the Poisson approximation. The comparison of the incidences between groups was carried out using Poisson model. The survival analysis was graphed using Kaplan-Meier curves.

### Ethical considerations and data confidentiality

This study has been approved by the Clinical Research Ethics Committee of the IDIAP Jordi Gol, including the waiver of obtaining informed consent for the pseudonymised data from the control group of non-attendees according to current regulations (ethical approval document 21/080-PCV). Informed consent was not required for participants in the group of non-attendees because the information obtained from the clinical health records was pseudonymised. Confidentiality was ensured by a coding system that blocked availability of any possible identifier to the researchers. Participants in the group of attendees received thorough information on the project and were asked to sign an informed consent and a statement of responsibility to participate in it, and provide permission to use their data within the context of the project.

This project was carried out in keeping with The World Medical Association’s Declaration of Helsinki, the Convention on Human Rights and Biomedicine regarding biomedical research (2005), and subsequent updates (Declaration of Helsinki, Fortaleza, Brazil, October 2013), as well as the Regulations 2016/679 of the European Parliament, the Council of 27 April 2017 on Data Protection (RGPD), and the applicable national regulations.

## Results

**Figure 1:**
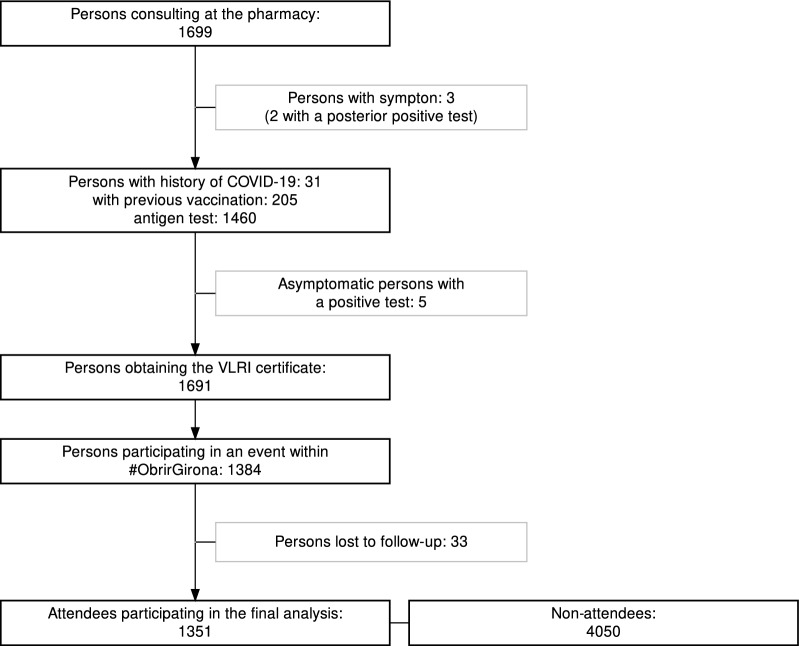
Flowchart of participation in the study.

During the study period, 1699 people tried to obtain the VLRT certificate at one of the participating pharmacies. Three of these individuals presented symptoms compatible with COVID-19 and were initially excluded (two of them had a posterior positive test, and one a negative test). Of the remaining ones, 31 (1.8%) had suffered COVID-19 confirmed with a positive PCR test (from three months before up to 15 days prior to the index date), 205 (12.1%) had been vaccinated, and the remaining 1460 underwent antigen testing. Five people within the latter group (undergoing antigen testing) tested positive. These five asymptomatic people, added to the above-mentioned two, who tested positive and had symptoms, amounted to seven individuals with a positive test result. Very low risk of transmission certificates were supplied to 1691 people. Of these, 1384 attended one of the events within the *ObrirGirona* project. The 14-day follow-up was attained for 1351 participants (97.6%), who were finally included in the analysis (Fig. 1). Even though three random controls (non-attendees) were selected for each person who attended an event, three of the attendees could only be matched to two controls (non-attendees); thus, the final numbers of analysed participants were 1351 people who attended an event (intervention group) and 4050 non-attendees (controls).

Table 1 compares the participants’ characteristics of both groups and counts the number of attendees per event type. The mean age of the study population was 31.1 years, and 53% of participants were women. Eleven per cent of the attendees reported that they had suffered from COVID-19 at some time since the beginning of the pandemic. Thirteen per cent of attendees had been vaccinated. Participants in both groups were similar regarding these characteristics, as expected when matching. The majority of participants in the intervention group attended the concerts: 86.4% of them; whereas 13.6% of attendees went for dinner at the restaurants.

**Table 1:** Number of attendees by event type and participants’ characteristics at index date (1 April–21 May 2021), Catalonia, (n = 5401).

	Intervention group	Control group	p-value
**Number of attendees; n (%)**			
Concerts	1167 (86.4%)	–	–
Restaurants	184 (13.6%)	–	–
**Total n**	1351	4050	
Women; n (%)	716 (53%)	2146 (53%)	1.00
Age; mean (SD)	31.1 (10.1)	31.2 (10.4)	0.68
History of COVID19 (self-reported); n (%)	146 (10.8)	436 (10.8)	1.00
Vaccinated people; n (%)	173 (12.8)	516 (12.7)	0.98

### Incidence of COVID19 during follow-up

The estimated incidences by group at seven and 14 days of follow-up are shown in Table 2. During the first seven days of follow-up, the incidence rate was lower in the intervention group (attendees), even though the difference between groups was not statistically significant.

**Table 2:** Incidence rates of SARS-CoV-2 infection by group at 7 and 14 days of follow-up, Catalonia, (n = 5401).

	Intervention group	Control group	p-value
n	1351	4050	
**Follow-up**			
**7 days (person-days)**	9456	28,336	
Cases	1	6	
Incidence rate (CI 95%)*	10.6 (0.3–58.9)	21.2 (7.8–46.1)	0.52
**14 days (person-days)**	18,904	56,625	
Cases	3	10	
Incidence rate (CI 95%)*	15.9 (3.3–46.4)	17.7 (8.5–32.5)	0.87

*Cases per 100,000 person-days.

**Figure 2:**
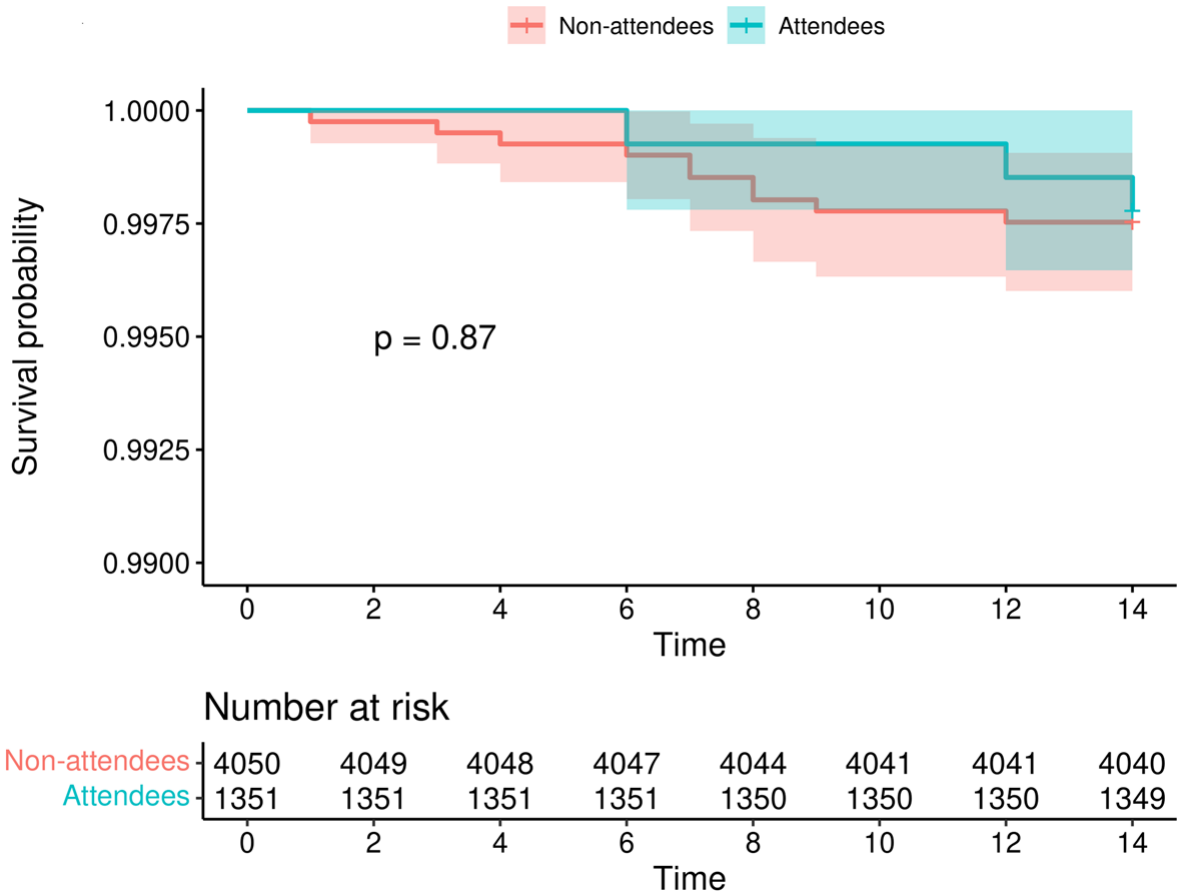
Kaplan–Meier survival curves by group during 14 days of follow-up (n = 5401).

During 14 days of follow-up, the incidence rate in the intervention group was 15.9 per 100,000 person-days, and the cumulative incidence was 222.1 per 100,000 persons; the corresponding figures in the group of non-attendees were 17.7 and 246.9, respectively. These differences were not statistically significant (as in the analysis with seven days of follow-up). The Kaplan–Meier survival curves are shown in Fig. 2.

### Report of the logistical and implementation challenges

Minor implementation problems arose only in 10% of cases, mainly because the users had not downloaded the app, or did not present adequate identification. Technical errors related to the app occurred in less than 0.5% of cases. All problems were readily solved.

### Satisfaction survey

A subsample of 148 people participated in a satisfaction questionnaire. Regarding overall satisfaction, 89.2% of respondents agreed or totally agreed with the process of obtaining the digital pass. As for the economic cost, 60.8% of respondents agreed or totally agreed that the cost was reasonable. Only 9.5% disagreed or totally disagreed. Finally, 83.1% of respondents agreed or totally agreed when asked if they would recommend following this process to a friend.

## Discussion

The results of our study showed that those people who attended public events with a certified digital pass did not have a higher SARS-CoV-2 infectious rate than a group that did not attend such events. The digital pass certified a negative antigen test result, or immunity against SARS-Cov-2 (confirmed vaccination or recovery from COVID-19). This risk screening strategy was grounded on the coordination of the primary care services and a community pharmacy network to ensure its feasibility and was well received by the participants in the events. Allowing safe social interactions not only would help alleviate the mental burden that lingering social restrictions inflict on the population, but it would also contribute to keeping the local economies open, and to the continuity of the cultural, sports, and catering sectors.

The observed cumulative incidences were similar to those in our area at the time of the study, which ranged from 103 to 362 per 100,000 persons (at 14 days) and had a median value of 309, supporting the validity of our results. Supplementary Fig. S1 shows the incidence of SARS-CoV-2 in our area during the study period^14^. Our findings also concur with previous reports. The PRIMACOV study^8^ included 1047 participants, aged 18–59 years, with no COVID-19 diagnosis in the 14 days previous to a concert held in Barcelona (Catalonia), and a negative antigen test result before entering the venue. Of those, 465 were randomly selected to go to the concert and 495 did not attend and comprised the control group. Both groups completed the follow-up visits. Results showed that none of the participants who went to the concert had a positive antigen test for SARS-CoV-2 during follow-up^8^. Conclusions of an observational subsequent study that included 5000 individuals, to test the preventive measures in a larger population, confirmed the lack of remarkable transmission events during a concert^10^. A very large randomised trial (SPRING) carried out in France aimed to compare nearly 4500 people assigned to attend an indoor live concert with over 2000 assigned not to attend it, and also found no increase in the SARS-CoV-2 transmission. In this trial, all participants underwent a screening test within the 3 days previous to the event, and around 7% of them had been vaccinated with two doses^9^. In our study, we used vaccination status and history of previous COVID-19, in addition to rapid antigen testing, to certify immunity against SARS-CoV-2, aiming to examine the available tools.

Rapid antigen tests have a high sensitivity to detect SARS-CoV-2, up to 98.6% when the viral load is high enough to suppose likely infectiousness (which could generally be considered under a cycle threshold of 30 if a PCR was performed)^14–16^. Rapid antigen tests in our study were not necessarily performed right before entering a concert, as in the PRIMACOV, but had a validity of up to 36 h before any event, because we were also aiming to examine the feasibility of implementation at a large scale. This was in line with the SPRING trial, where participants were tested within the 3 days before the event. Further studies to determine the maximum acceptable gap of time from the performance of rapid antigen test until the event would be of interest.

Only a minority of participants presented a certification of vaccination status as proof of immunisation in the digital pass. However, percentages shifted, later on, towards the predominance of fully vaccinated people. The overall effectiveness for the prevention of infection has been estimated at 91% for fully vaccinated people^17^. Vaccination not only poses a lower risk of SARS-CoV-2 infection, but also attenuates the effects of breakthrough infections^17–19^, which is of paramount importance within the context of a wave.

History of recovery from SARS-CoV-2 infection was the third condition that justified a low risk of SARS-CoV-2 transmission^20–22^. A recent review reported a very low probability of detecting replication-competent virus from samples of people with SARS-CoV-2 infection in cultured cells after 8–15 days from symptom onset^20^. Within this range, the duration of infectivity was longer in samples from individuals with a more severe illness^20^. Epidemiological data also reflected these results^23^.

Another novelty in our study is the type of activities considered to evaluate SARS-CoV-2 transmission. Additional to concerts or other mass-gathering events where participants used the mask, we also included dinners at restaurants, a context in which people are at a relatively close distance without covering their faces while eating. At the time of the study, mask and security distance was mandatory indoors and outdoors, along with adequate ventilation of indoor public spaces, access control of attendees to cultural and leisure activities, use of hand sanitiser at the entrance of indoor public spaces, social distance whenever possible, and control of mobility indoors, amongst a list of preventive measures and indications detailed in Supplementary Table S1^24,25^.

Finally, the most relevant addition concerned the organisational structure. The PRIMACOV study reported the difficulty of testing thousands of people within a few hours before the concert^8^. Striving to address this question, we considered the type of sample and participation of a pharmacy network. The rapid antigen tests were performed from a nasal swab, which is easier to obtain than the nasopharyngeal swab, and thus facilitated supervised self-administration, which in turn allowed testing for a higher number of individuals in a given period of time. Rapid antigen tests from a self-administered nasal swab under professional supervision have similar sensitivity to those from a nasopharyngeal swab^26,27^. On the other hand, the adequacy of the spaces where the COVID-19 VLRT certificate could be obtained is important. These spaces needed to meet certain quality and safety requirements, regarding logistics, training and protection of the staff involved, and protection of all participants. Pharmacies are accessible premises spread throughout the territory and thus good candidates to perform the necessary tests and checks to obtain a digital pass, provided they comply with the appropriate quality and safety measures. Participating pharmacies had to allocate a specific space for this purpose that was isolated, separated from the other customer service areas, and with adequate ventilation. Such space was duly cleaned after each sample was taken.

Another key point of the organisation was the technology to frame the certification process in a safe and efficient manner. We used the AOKpass digital app, (developed by the ICC, International SOS, and SGS), to hold a safe, easy-to-use pass that certified a VLRT condition in an access control that could be performed by a non-healthcare professional. The VLRT condition implied vaccination against SARS-CoV-2, recovery from COVID-19, or a negative result in an antigen test. This type of certification would be analogous, or could even be adapted for its use as a certification to travel throughout Europe.

The following limitations should be considered in our study. First, the group of attendees and non-attendees could be different regarding certain variables omitted in the matching. However, we considered the main determinants of SARS-CoV-2 infection and used methodological matching^28,29^. Second, Howthorne bias is intrinsic to trials: participants are aware that they are part of an experiment and thus certain modification in their behaviour is bound to occur, which has to be considered when applying the results beyond the study context. Finally, compliance with safety measurements might be lower in participants lost to follow-up, which could partly bias the results.

Lifting of restrictions on social contacts resulted in an increase in cases associated to mass gathering events, especially in non-vaccinated populations. Increasing vaccination rates contributed to reducing the severity of infected people, but the more vulnerable were eventually also affected -including those with contraindication for vaccination- due to the high transmission capacity of this virus, which could be noticed in the number of hospitalisations and severe cases^30^.

We examined the feasibility of the implementation of a certificate that could be obtained through a process that involves the coordination of the primary care services and a network of already-existing pharmacy offices adapted for such purpose. Our study shows that the reopening of social life could be done in a controlled way, using an app to certify the minimised risk of SARS-CoV-2 transmission. Such an approach could benefit the wellbeing of the population, particularly regarding mental health, and help socio-economic sectors like culture, sports, and catering.

## Conclusion

Attendance to events that involved social interaction with a certified digital pass was not associated with an increased rate of SARS-CoV-2 infection compared to a control group who did not attend these events. Such certifications stated a negative result in an antigen test or immunity against SARS-CoV-2, through vaccination or recovery from COVID-19. Logistical problems during the process of obtaining the certificate were very infrequent and easy to solve. The users’ overall opinion on the accreditation process was satisfactory or very satisfactory for the vast majority of participants. The implementation of the presented approach is feasible and could allow socio-economic spaces and activities to remain open.

## Supplementary Information

Supplementary Information.

## Data Availability

The datasets analysed in this study are not publicly available due to legal reasons related to data privacy protection but are available from the corresponding author on reasonable request.
