# Supplementary material for: SARS-CoV-2 transmission risk screening for safer social events: a non-randomised controlled study
Source: Sci Rep. 2022 Jul 27;12:12794. doi: 10.1038/s41598-022-16905-w (PMC9326423; doi:10.1038/s41598-022-16905-w)
Supplement: Supplementary file 1 — Supplementary Information. [file 41598_2022_16905_MOESM1_ESM.docx]

**SARS-CoV-2 transmission risk screening for safer social events: a non-randomised controlled study**

Rafel Ramos*^1,2,3^, Lia Alves-Cabratosa^1^, Jordi Blanch^1^, Àlex Pèlach^4^, Laura Albert^5^, Quirze Salomó^4^, Sílvia Cabarrocas^5^, Marc Comas-Cufí^1,6^, Ruth Martí-Lluch^1,3^, Anna Ponjoan^1,3^ Maria Garcia-Gil^1^, Salomé de Cambra^4^, Albert d’Anta^4^, Elisabet Balló^2,7^, Albert Alum^7^, Rosa Núria Aleixandre^5,8,9^.

^1^Vascular Health Research Group (ISV-Girona), Institut Universitari d’Investigació en Atenció Primària Jordi Gol (IDIAP Jordi Gol), Girona, Catalonia, Spain.

^2^Department of Medical Sciences, School of Medicine, Campus Salut, Universitat de Girona, Girona, Catalonia, Spain.

^3^Biomedical Research Institute of Girona (IdIBGi), Salt, Girona, Catalonia, Spain.

^4^Centre Blockchain de Catalunya, Parc Científic i Tecnològic de la Universitat de Girona, Girona, Catalonia, Spain.

^5^Col.legi de Farmacèutics de Girona, Girona, Catalonia, Spain.

^6^Computer Science, Applied Mathematics and Statistics department, Universitat de Girona.

^7^Unitat de Qualitat i Seguretat del pacient, Atenció Primària, Institut Català de la Salut, Girona, Catalonia, Spain.

^8^Consell de Col.legis Farmacèutics de Catalunya, Barcelona, Catalonia, Spain.

^9^Consell Social Universitat de Girona, Girona, Catalonia, Spain.

**Corresponding Author:**

Rafel Ramos Blanes

ISV Girona – IDIAPJGol

c/ Maluquer Salvador, 11 baixos

17002 Girona (Catalonia, Spain)

Telephone number: +34 972 487968

Email address: [rramos.girona.ics@gencat.cat](mailto:rramos.girona.ics@gencat.cat)

# Supplementary Table 1. Summary of local restrictions in force (1 April-21 May 2021), Catalonia^1,2^

| Social activities | maximum of 6 persons | | Meetings in indoor spaces: restricted as much as possible and limited for vulnerable persons; within the social bubble. | | |
| --- | --- | --- | --- | --- | --- |
| Confinement in the area | Catalonia: entry and exit restricted unless justified reason. | | County: mobility to another county limited to members of the same coexistence bubble. Teleworking mandatory whenever possible. | | |
| Curfew | From 22:00h to 06:00h. | | Except: justified causes. | | |
| Work | Teleworking whenever possible | | Suspended: congresses, conventions, fairs and similar activities | | |
| Education | University: up to 30% of capacity | | High school and equivalent: distance learning | | Primary school: in-class activities |
| Extra scholar activities | Groups of maximum 6 persons or social bubble. | | Trips including overnight stay allowed within the social bubble. | | |
| Sports | Outdoors: 50% | Indoors 30%. Where ventilation could not be reinforced mask was compulsory. Reopening of changing rooms. | Competitions:  With public: 50% outdoors; 30% indoors.  Without public: professional football and basketball. | | |
| Shops and shopping centres | Opening hours: 6:00h to 21:00h, including weekends.  Up to 30% of capacity | | |  | |
| Restaurants | Opening hours: 7:30h-17:00h  Outdoors: 100% of capacity  Indoors: 30% | Maximum 4 persons per table. | 2 m separation between diners or tables. | | Take away until 22:00h. Delivery until 23:00h. |
| Cultural and leisure activities | 50% museums, libraries, and galleries. | Cultural and leisure activities, including concert halls and similar venues. Maximum 500 seated persons. With reinforced ventilation maximum 1,000 persons. | Playgrounds, up to 50% of capacity; until 20:00h | | Gaming arcades, casinos, bingos, max 100 persons. With reinforced ventilation max 250 persons. Groups max 6 persons, with a distance of 2 metres between groups.  Closed: complementary services of bar and restaurant. |
| Religious events | 30%of maximum capacity, maximum 500 persons. Outdoors and with optimum ventilation, maximum 1000 persons. | | | | |

# Supplementary Figure 1. Cumulated incidences (per 100,000 persons) at 14 days in the area of Girona (Catalonia), (1 April-21 May 2021) 3**
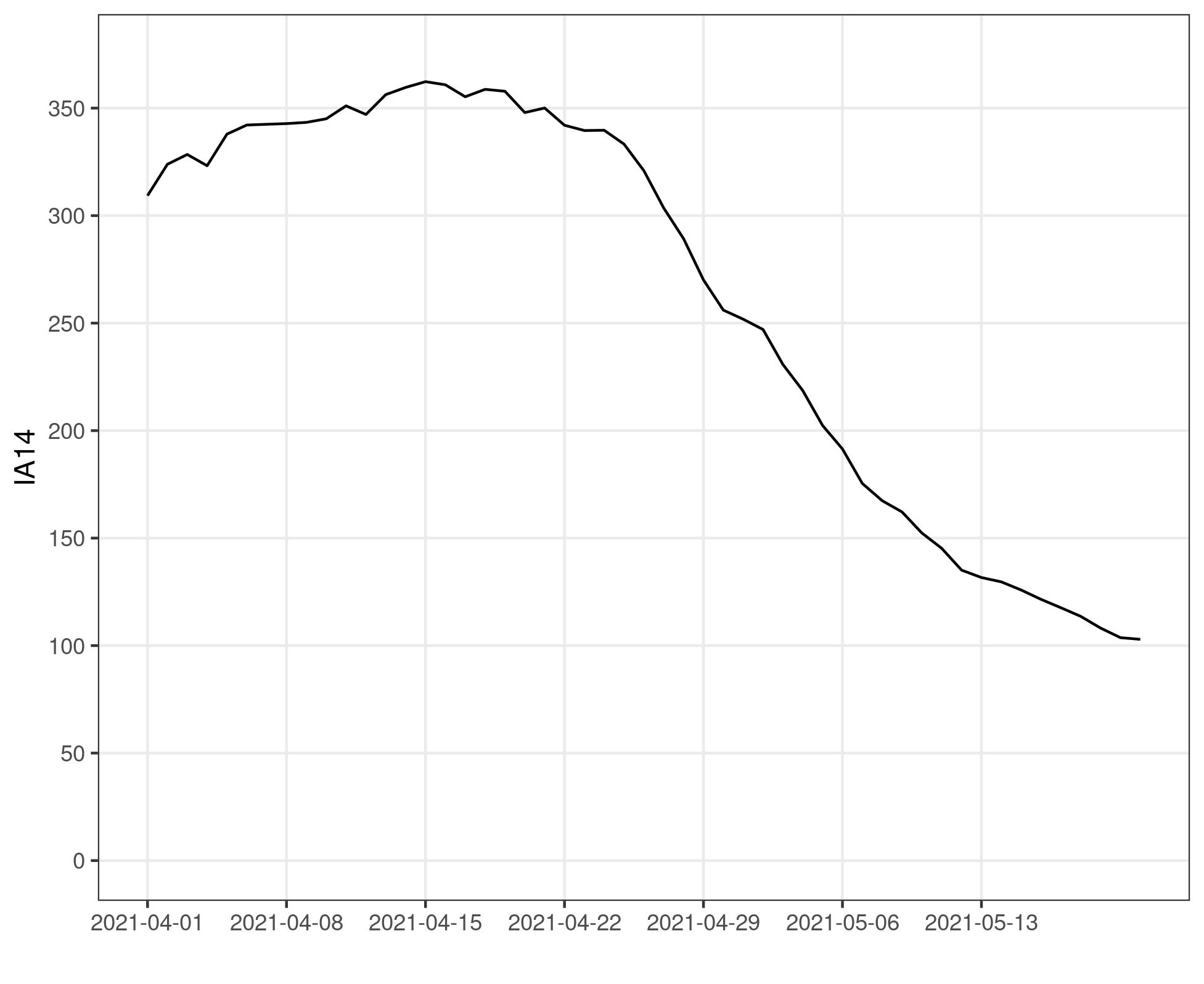
**

# **References**

1. Departament de Salut Generalitat de Catalunya. *RESOLUCIÓ SLT/1088/2021, de 16 d’abril. DOGC*. https://dogc.gencat.cat/ca/document-del-dogc/?documentId=896691 (2021).

2. Departament de Salut Generalitat de Catalunya. *RESOLUCIÓ SLT/845/2021, de 26 de març. DOGC*. https://dogc.gencat.cat/ca/document-del-dogc/?documentId=896691 (2021).

3. CataluDepartamentnya, de S. G. de. Dades COVID SETMANAL. Girona. https://dadescovid.cat/setmanal?tipus=regio&codi=64&id_html=ambit_7.
